# Supplementary figures and images for: Rapid assembly of functional modules for generating human artificial chromosome constructs compatible with epigenetic centromere seeding
Source: Chromosome Res. 2025 Dec 1;33(1):29. doi: 10.1007/s10577-025-09788-w (PMC12669295; doi:10.1007/s10577-025-09788-w)

**Figure S1**

**A**

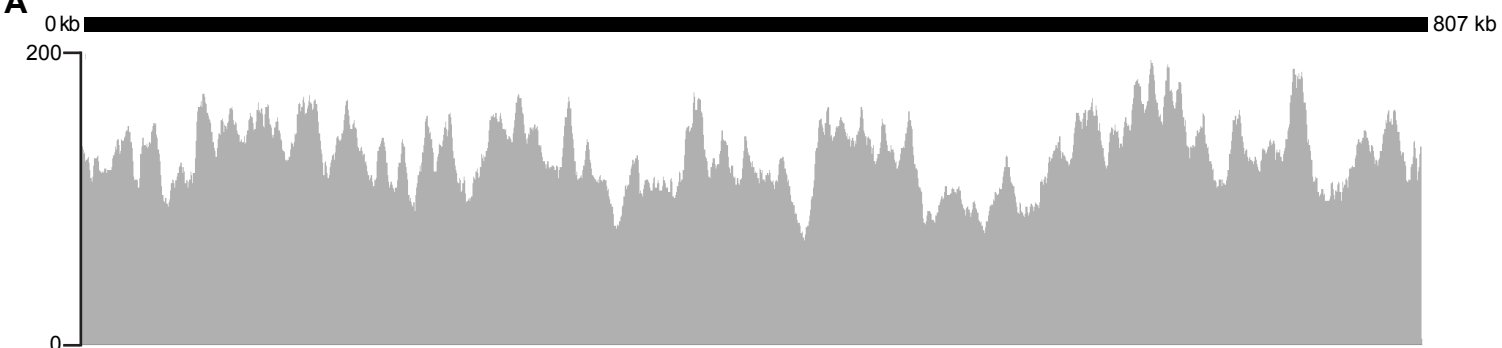

**B**

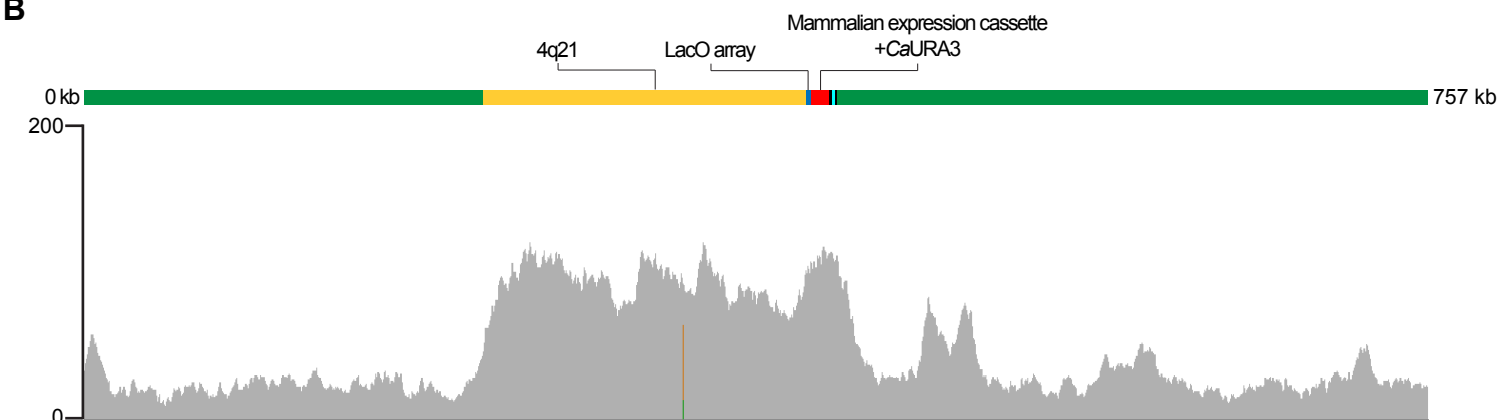

Supplement: Supplementary file 1 — Supplementary file1 M. mycoides stuffer DNA is under-represented in sequencing libraries of recombinant yeast strains. A) An example Integrated Genomics Viewer alignment track showing coverage throughout the de novo-assembled native chromosome 2. B) Integrated Genomics Viewer alignment track showing raw reads mapped to de novo-assembled YAC-Mm-4q21Short LacO again reveals relative underrepresentation of prokaryotic stuffer DNA (PDF 298 KB) [file 10577_2025_9788_MOESM1_ESM.pdf]

Figure S2

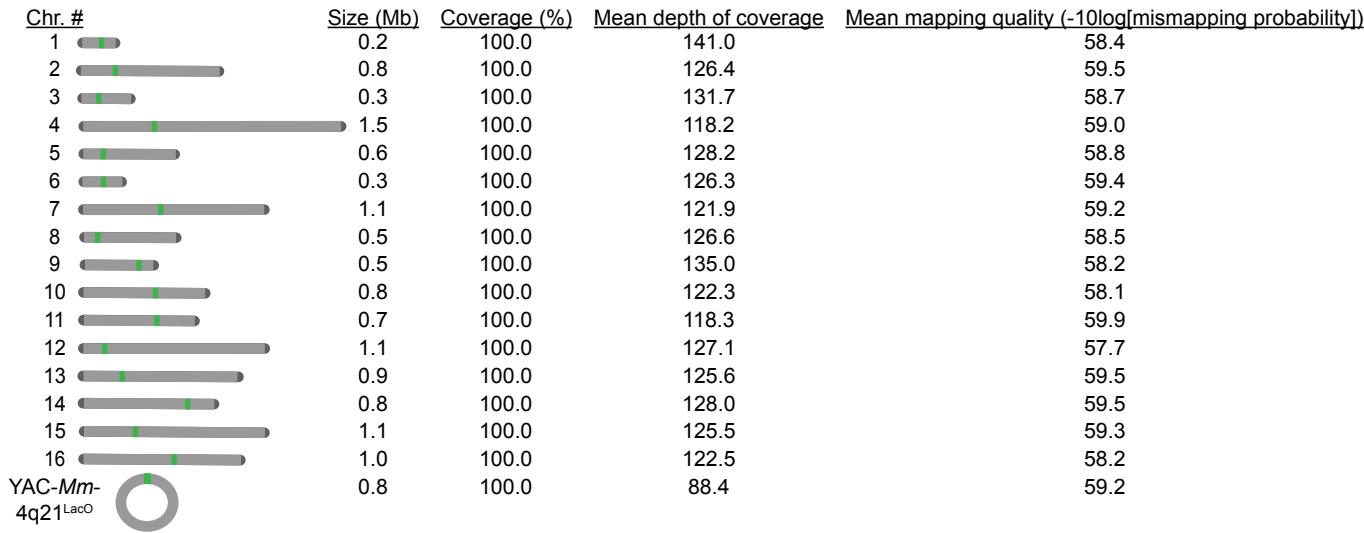

Supplement: Supplementary file 2 — Supplementary file2 Detailed summary statistics of assembly quality. Graphical depiction of the de novo-assembled YAC-Mm-4q21LacO along with statistics reflecting quality of assembly. Coverage denotes the percent of assembled chromosomes covered by reads. Mean depth of coverage denotes the average number of times that constituent nucleotides of a chromosome are covered by reads. Note that the YAC-Mm-4q21LacO exhibits reduced mean depth of coverage, which is attributable to the prokaryotic stuffer (see Results). Mean mapping quality denotes the average probability that reads assigned to the chromosome are mismapped and is reported on a scale of 0-60.0 with higher values representing lower mismapping probability (i.e., 60.0 = 1 mismapped read per 1 x 10^6^ mapped reads) (PDF 288 KB) [file 10577_2025_9788_MOESM2_ESM.pdf]
